# Transcriptional and post-transcriptional regulation of young genes in plants

**DOI:** 10.1186/s12915-022-01339-7

**Published:** 2022-06-09

**Authors:** Vivek Kumar Raxwal, Somya Singh, Manu Agarwal, Karel Riha

**Affiliations:** 1 Department of Botany, University of Delhi, Delhi, 110007 India; 2 Central European Institute of Technology (CEITEC), Masaryk University, Brno, Czech Republic

**Keywords:** Abiotic stress, Evolutionary capacitance, Nonsense-mediated RNA decay, Open chromatin, Orphan genes, Young genes

## Abstract

**Background:**

New genes continuously emerge from non-coding DNA or by diverging from existing genes, but most of them are rapidly lost and only a few become fixed within the population. We hypothesized that young genes are subject to transcriptional and post-transcriptional regulation to limit their expression and minimize their exposure to purifying selection.

**Results:**

We performed a protein-based homology search across the tree of life to determine the evolutionary age of protein-coding genes present in the rice genome. We found that young genes in rice have relatively low expression levels, which can be attributed to distal enhancers, and closed chromatin conformation at their transcription start sites (TSS). The chromatin in TSS regions can be re-modeled in response to abiotic stress, indicating conditional expression of young genes. Furthermore, transcripts of young genes in *Arabidopsis* tend to be targeted by nonsense-mediated RNA decay, presenting another layer of regulation limiting their expression.

**Conclusions:**

These data suggest that transcriptional and post-transcriptional mechanisms contribute to the conditional expression of young genes, which may alleviate purging selection while providing an opportunity for phenotypic exposure and functionalization.

**Supplementary Information:**

The online version contains supplementary material available at 10.1186/s12915-022-01339-7.

## Background

The advent of whole-genome sequencing led to the discovery of a subset of new genes in all domains of life that lack homologs in other lineages [1–4]. These genes, also called orphan or evolutionary young genes, may arise from pre-existing genes by diverging until no homology remains, or they may be born de novo from non-coding DNA [5–8]. In contrast to old genes, young genes are short, rapidly evolving, and usually do not have essential functions. They are therefore under weaker positive selection [9, 10]. Furthermore, the production of non-functional proteins from young genes may represent an energetic burden for the cell, and their evolutionarily non-optimized structure can lead to non-productive interactions, some of which may interfere with cellular functions [11, 12]. Consequently, despite increasing the population’s genetic diversity, most young genes are rapidly lost either due to genetic drift or purging selection, and only a few are fixed in the genome [13]. This raises the question of whether some young genes have the means to hide from purifying selection, expanding their lifespan in a genome and thereby increasing their chances of acquiring novel functions.

One possibility to mitigate the effect of purifying selection is by limiting the extent of expression and/or conditioning it by developmental or environmental cues. Indeed, young genes often have low expression due to the deposition of repressive chromatin marks and the lack of well-developed *cis*-regulatory elements required for transcription [14, 15]. The low expression level of young genes can lessen the burden of protein misfolding, hence reducing the negative selection pressure [11, 12]. Further, spatial or temporal restriction of expression may expand the lifespan of young genes by avoiding untimely exposure to natural selection and may provide an opportunity for phenotypic manifestation and functionalization. In nematodes, young genes are born in the vicinity of enhancers and utilize their *cis*-regulatory elements to express themselves in limited cell types and tissues [16]. Moreover, the permissive transcription environment of isolated compartments, such as the testes in animals and pollen grains in plants, provides a perfect breeding ground for young genes to mature and gain function [17, 18].

In this study, we investigated the mechanisms that contribute to the low or conditional expression of young genes via transcriptional and post-transcriptional regulation in rice and *Arabidopsis*. We propose that the restricted expression of young genes through these mechanisms can mitigate exposure to negative selection and offer an opportunity for phenotypic manifestation under certain conditions, which permits gene functionalization and genetic fixation in a population.

## Result and discussion

### Young genes are associated with closed chromatin and distal enhancers

**Fig. 1:**
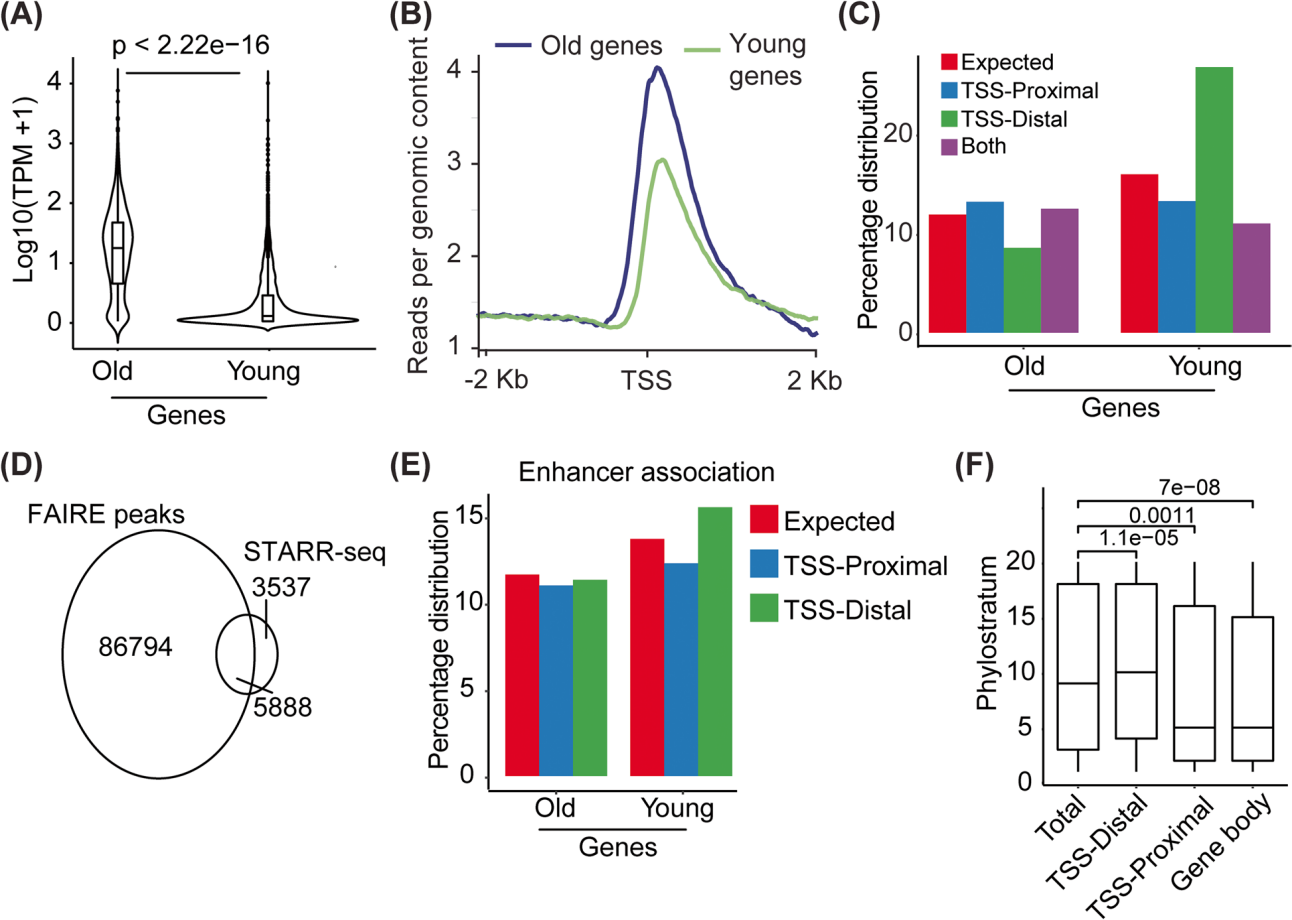
Transcriptional regulation of the young genes. **A** A violin plot showing the expression of old and young genes. The expression of genes in transcript per million (TPM) was log10-transformed before plotting. The statistical significance of the difference was calculated using the Mann-Whitney test. **B** A line plot showing normalized coverage of FAIRE-seq reads around the transcription start site (TSS) of old (PS1) and young (PS19) genes. **C** A bar plot was drawn to show the enrichment of FAIRE-seq peaks at either the TSS-proximal (up to 1.5 kb upstream of TSS), TSS-distal (> − 1.5 kb from TSS), or both (FAIRE-seq peak present at TSS-proximal as well as TSS-distal intergenic regions) of old and young genes. Enrichment was higher than background (expected) and was calculated as the total percentage of genes present in the old or young gene categories. **D** An area proportional Venn diagram showing the overlap of FAIRE-seq-identified open chromatin regions and STARR-seq-identified enhancers. **E** A bar plot was drawn to show enrichment of enhancers at the TSS-proximal (up to 1.5 kb upstream of TSS) and TSS-distal (> − 1.5 kb from TSS) regions of old and young genes. The enrichment was seen over the background (expected), calculated as the total percentage of enhancers present in the old or young gene categories. **F** A box plot showing the association of STARR-seq-identified enhancers with the age of genes. Enhancer is characterized as TSS-distal if it is present > 1.5 kb upstream of the TSS, whereas TSS-proximal enhancers are located within 1.5 kb upstream of TSS. If an enhancer is present at any part of the gene body, then it is characterized as a gene body enhancer. The age of the nearest gene is plotted at the *Y*-axis, where phylostratum 1 denotes the oldest and phylostratum 19 denotes the youngest class of genes

We performed a protein-based homology search across the tree of life to determine the evolutionary age of protein-coding genes present in the rice genome using Phylostratr [19]. The genes were grouped into phylostrata (PS) according to their evolutionary age, such that PS1 contains genes with the oldest known ancestor homolog, whereas the last phylostrata (PS19) includes the evolutionary youngest genes with no known ancestral homolog (Fig. S1). Consequently, PS1 was enriched with genes involved in biological processes such as replication, transcription, translation, cell cycle, meiosis, and DNA repair. In contrast, no specific biological process categories were enriched in PS19. To understand the differences in the gene regulation and the underlying reasons, we compared the steady-state expression of evolutionary old (PS1) and young genes (PS19) and observed that young genes largely had low expression relative to the old genes (Fig. 1A).

Young genes have been reported to possess heterochromatic epigenetic signatures and a closed chromatin state in *Drosophila*, nematodes, and *Arabidopsis* [14, 20, 21]. To investigate whether the lower expression of young genes in rice was due to the closed chromatin architecture of their regulatory regions, we employed formaldehyde-assisted isolation of regulatory elements (FAIRE-seq) (Fig. S2; Additional file 2: Table S1, S2). We observed that the TSS-proximal regions of the young genes have a more closed chromatin conformation relative to old genes (Fig. 1B). Moreover, we observed an increased association of transposons with the TSS-proximal region of young genes in contrast to old genes (Fig. S3A). This is in agreement with higher levels of repressive histone modifications (H3K9me2 and H3K9me1) and lower levels of permissive modifications (H3K9ac and H3K4ac) at the TSS of young genes compared to old genes (Fig. S3B-E). These results suggest that a generally closed chromatin conformation limits the expression of young genes in rice.

Interestingly, our FAIRE-seq analysis in rice revealed an enrichment of open chromatin at the distal intergenic regions (> 1500 bp upstream from TSS) of young genes (Fig. 1C), which has also been observed in *Arabidopsis* (Fig. S4). Open chromatin at a distal intergenic region is indicative of an enhancer [22], and enhancers have been suggested to be involved in the birth of young genes [14, 16]. To determine whether distal upstream regions of young genes indeed contain enhancers, we determined the overlap between open chromatin, which we identified by FAIRE-seq, and enhancers, identified by STARR-seq, in rice [23]. Our overlap analysis revealed that more than 60% of the known enhancers overlapped with open chromatin regions (Fig. 1D). Next, we compared the enrichment of enhancers in TSS-proximal and TSS-distal regions. We found that enhancers are enriched in the TSS-distal regions of young genes but not in the TSS-distal regions of old genes (Fig. 1E). We also observed that the average age of the nearest gene to distal intergenic enhancers is younger in contrast to the enhancers present in the TSS-proximal region (Fig. 1F). Interestingly, we did not detect an enrichment of enhancers in TSS-proximal regions of young genes. This is in contrast to previous findings in nematodes which suggested that genes are born within open regions of enhancers [14]. Instead, our result suggests that, at least in rice, distally positioned enhancers play a regulatory role by conditioning the expression of young genes [24, 25]. The utilization of distal enhancers likely contributes to the lower expression observed for young genes, which reduces the cost to the cell of translating misfolded and possibly toxic proteins. Moreover, enhancers evolve faster than promoters, thereby providing an opportunity for young genes to evolve together with enhancers as they acquire novel transcription regulatory networks over time [26, 27].

### Abiotic stress alters chromatin architecture at TSS of young genes

**Fig. 2:**
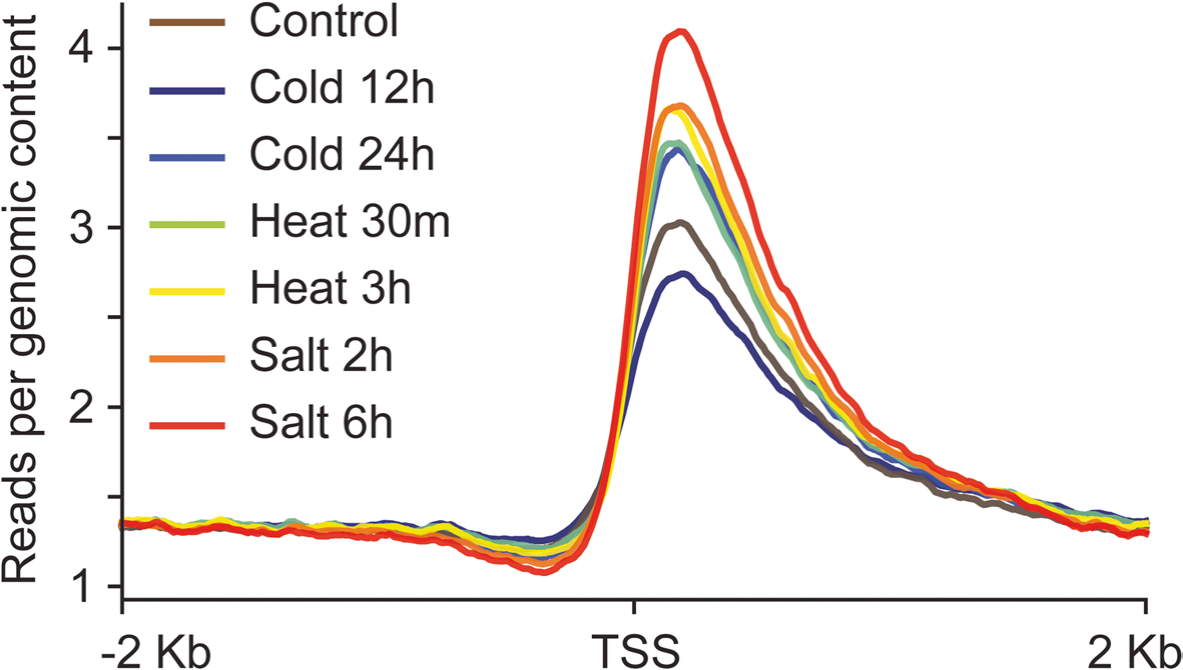
Abiotic stress re-models the chromatin architecture of young genes. A line plot showing the normalized coverage of FAIRE-seq reads around the TSS (± 2000 bp) of young genes in control and stress subject rice seedlings

The expression of young genes can provide species-specific adaptation to environmental challenges [15, 28–30], suggesting that their chromatin architecture and transcription are responsive to external stimuli. Since many young genes in rice have a closed chromatin conformation in the promoter-proximal regions, we investigated whether abiotic stresses have the potential to affect chromatin conformation. We performed FAIRE-seq on rice seedlings subjected to varying durations of cold, heat, and salt stress (see the “Methods” section). Except for cold stress (12 h), all of the abiotic stresses we tested increased chromatin accessibility around the TSS of young genes (Fig. 2). These results suggest that chromatin architecture can be re-modeled upon exposure to external environmental factors, allowing young genes to gradually evolve interactions between *cis*-regulatory elements and regulatory proteins, thereby providing an opportunity to increase their expression and gain function.

### Young gene transcripts are targeted by nonsense-mediated RNA decay

**Fig. 3:**
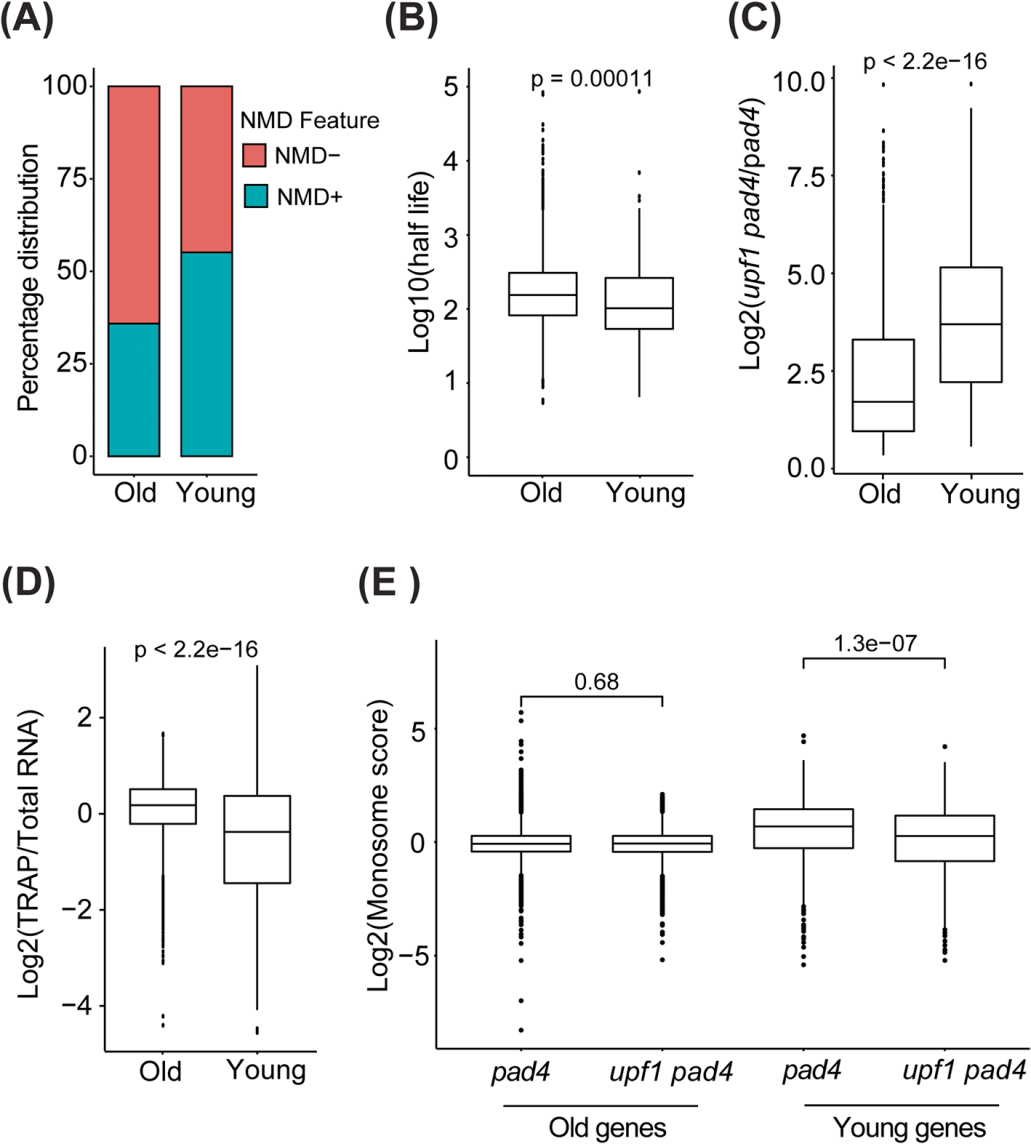
Nonsense-mediated RNA decay (NMD) post-transcriptionally regulates the abundance of young genes. **A** The accumulation of NMD features in old and young genes is presented as a bar plot. **B** A box plot representing the half-life (log10-transformed) of the old and young genes. **C** The relative change in expression of old and young genes due to UPF1 deficiency. **D** The association of old and young genes with the ribosome is shown as a box plot. **E** A box plot depicting the association of old and young genes with monosome and polysome either in *pad4* or UPF1-null (*upf1 pad4*)

Apart from transcriptional regulation, the expression of young genes can be reduced by post-transcriptional regulation. Young genes in *Drosophila* have been associated with increased occurrence of premature translation termination codons (PTCs) [31]. Since PTC-containing transcripts are degraded by the nonsense-mediated RNA decay (NMD) pathway [32, 33], we hypothesized that the expression of young genes is affected by NMD. To examine this hypothesis, we took advantage of the genetic and genomic resources available in *Arabidopsis* [34]. Indeed, compared to older genes, young genes were enriched for PTCs and long 3′UTRs, both hallmark NMD features (Fig. 3A). We also observed an increased incidence of PTCs in young genes of maize and *Arabidopsis* (Fig. S5). We further found that young genes have significantly reduced transcript stability compared to old genes (Fig. 3B). The *Arabidopsis upf1 pad4* mutant, which is severely compromised in NMD [34, 35], exhibits a more pronounced increase in the expression of young genes than old genes (Fig. 3C). These results suggest that young genes are subject to post-transcriptional regulation by NMD.

The increased targeting of young gene mRNAs by NMD implies lower levels of translation. Therefore, we next evaluated the relative efficiency of translation by scoring the differences in ribosome association between old and young genes using the Targeted Purification of Polysomal mRNA (TRAP) dataset [36]. We observed that transcripts from older genes had substantially higher levels of ribosome association than transcripts from younger genes, indicating less efficient translation of young genes (Fig. 3D). Previously, we determined that inactivation of NMD leads to increased translation of NMD-targeted transcripts, which was manifested as a shift of the transcripts from monosomal to the polysomal fraction of ribosomes [34]. If NMD contributes to the translational repression of young genes, we also anticipated a similar trend for young gene transcripts. Indeed, NMD inactivation led to a significant decrease of the monosome score for young transcripts, which indicates their shift to polysomes and increased translation (Fig. 3E). In contrast, no significant difference was observed in the monosome score of older genes in the presence or absence of UPF1 (Fig. 3E). These results further substantiate the role of NMD in limiting the expression of young genes at the level of RNA stability and translation. Because NMD efficiency changes in response to environmental and developmental cues [37, 38], the repression of young genes by NMD is not constitutive. Rather, it could be lifted under certain conditions. The observation that young genes are subject to regulation by NMD is in line with our hypothesis where we proposed that NMD can act as an evolutionary capacitor, permitting the accumulation of cryptic genetic variation and exposing it conditionally to the selection [37].

## Conclusions

We propose that young genes tend to have low expression levels due to closed chromatin, limited transcription by enhancers, and post-transcriptional degradation by NMD to avoid their untimely exposure to purifying selection. All three of these mechanisms are responsive to environmental stress and developmental signals, which provide an opportunity for the conditional expression and phenotypic manifestation of young genes, a prerequisite for gene functionalization.

## Methods

### Plant material, growth conditions, and stress treatment

*Oryza sativa* L. Japonica nipponbare was grown in a plant growth chamber (Conviron®) at 28 °C under a photoperiod of 16 h/8 h. Fourteen-day-old seedlings were subjected to heat (42 °C for 30 min and 3 h), cold (4 °C for 12 h and 24 h), and salt (250 mM NaCl for 2 h and 6 h) stress. The 14-day-old seedlings grown at 28 °C were taken as the control sample.

### Formaldehyde-assisted isolation of regulatory elements (FAIRE-seq)

FAIRE-seq was performed on two independent replicates of 14-day-old stress-treated and control seedlings as per the protocol described previously [39]. The raw sequencing reads were aligned to the rice reference genome (IRGSP 1.0) using Bowtie2 with default parameters [40]. The reads aligning to the region of the chromosome with a known insertion site of the mitochondrial and chloroplast genome were removed. To remove reads mapped to multiple positions on the genome, reads with a mapping score of less than 10 were filtered out. To remove potential PCR duplicates, reads with the same start and end positions were considered only once. Broad peaks were called by MACS2 with default parameters except the no-model option to identify open chromatin regions in the rice genome [41]. Furthermore, peaks having a read count < 1 RPM in any biological replicates were removed from the analysis. A scatter plot was generated to observe the reproducibility of peaks among biological replicates. The overlap analysis was performed using the R package ChIPpeakanno [42].

### RNA-seq and analysis

RNA-seq libraries from RNA extracted (the Qiagen plant RNA extraction kit) from two independent replicates were prepared using the TruSeq RNA sample preparation kit (Illumina Inc., USA). The libraries were sequenced for 50-bp single-end sequencing on Illumina’s HiSeq 2000 platform. The sequencing reads were filtered for quality using Trimgalore (https://www.bioinformatics.babraham.ac.uk/projects/trim_galore/), and high-quality reads were pseudo aligned to Rice transcriptome (ensemble 46 version) using Kallisto version 0.46.0 with default parameters [43]. The differential expression analysis was performed using a limma-voom pipeline enabled in 3D-RNA-seq [44].

### Evolutionary age classification of genes, NMD features, and mRNA half-life analysis

The evolutionary age or phylostratum of each peptide present in the Rice (*Oryza sativa* Nipponbare version), maize (B73 RefGen_v4), and *Arabidopsis* (Araport 11) was determined using Phylostratr [19]. In brief, a pairwise BLAST of the proteome of focal species (rice, *Arabidopsis*, and maize) against each of the species present in the NCBI tree of life (uniport proteome) was performed. The best hit for each protein of focal species was extracted and assigned phylostratum associated with the deepest clade. The subset of genes with no inferred homologs was classified as the evolutionary youngest genes. A transcript is defined as having an NMD feature if it has a premature termination codon (PTC) before (greater than 50 bp) the last exon junction complex (EJC) or the length of its 3′UTR exceeds 350 bp. The mRNA half-life, TRAP, and NMD data were taken from [34, 36, 45].

## Supplementary Information

**Additional file 1: Figure S1**. Phyllostratographic classification of rice genes **Figure S2**. FAIRE-seq identifies reproducible peaks. **Figure S3**. TSS of young genes associates with transposons and repressive histone modification signatures. Data from [47]. **Figure S4**. A bar plot showing the percentage distribution of FAIRE-peaks in either the TSS proximal (<-1.5kb of TSS) or TSS distal region (> -1.5kb of TSS) of old and young genes of *Arabidopsis thaliana*. **Figure S5**. A bar plot showing the distribution of premature translation termination codons (PTC) in old and young genes of Arabidopsis, rice, and maize.

**Additional file 2: **NGS data. **Table S1**. Mapping percentage of FAIRE-seq and RNA-seq data. **Table S2**. Expression of young and old genes in rice seedlings.

## Data Availability

All the sequencing data have been submitted to the Gene Expression Omnibus (GEO) repository under the accession number GSE192747 [46].
