# Supplementary material for: Transcriptional and post-transcriptional regulation of young genes in plants
Source: BMC Biol. 2022 Jun 9;20:134. doi: 10.1186/s12915-022-01339-7 (PMC9178820; doi:10.1186/s12915-022-01339-7)
Supplement: Supplementary file 1 — Additional file 1: Figure S1. Phyllostratographic classification of rice genes Figure S2. FAIRE-seq identifies reproducible peaks. Figure S3. TSS of young genes associates with transposons and repressive histone modification signatures. Data from [47]. Figure S4. A bar plot showing the percentage distribution of FAIRE-peaks in either the TSS proximal (<-1.5kb of TSS) or TSS distal region (> -1.5kb of TSS) of old and young genes of Arabidopsis thaliana. Figure S5. A bar plot showing the distribution of premature translation termination codons (PTC) in old and young genes of Arabidopsis, rice, and maize. [file 12915_2022_1339_MOESM1_ESM.docx]

**Additional File 1: Supplementary information**

**Figure Tittle and Legends**

**Fig. S1. Phyllostratographic classification of rice genes.**

A protein-based homology search across the tree of life to determine the evolutionary age of protein-coding genes present in the rice genome was performed using Phylostratr. A bar plot was drawn that represents the percentage of genes present in each inferred age group or phylostratum. PS1 represents the oldest subset of genes, whereas PS19 represents the youngest genes.

**Fig S2: FAIRE-seq identifies reproducible peaks.**

(A-I) A scatter plot showing the reproducibility of FAIRE-seq identified regions among two biological replicates of control and stress subjected samples. Each dot represents the log 10 normalized read per million values of one FIR in biological replicate 1 (x-axis) and replicate 2 (y-axis). The R-value is the Pearson correlation coefficient, and the low p-value rejects the null hypothesis that the coefficient is equal to zero. The line represents the linear regression fit. (J) A principal component analysis plot representing variation in the FAIRE-seq identified peaks between stress and control sample. More than 90% of the variation can be described by principal components 1 and 2

**Fig. S3. TSS of young genes associates with transposons and repressive histone modification signatures**

**(A)** TSS proximal region of young genes shows the increased accumulation of transposons compared to old genes **(B-E)** A line plot showing the deposition of various histone modification marks over the TSS of young and old genes.

**Fig. S4.** A bar plot showing the percentage distribution of FAIRE-peaks in either the TSS proximal (<-1.5kb of TSS) or TSS distal region (> -1.5kb of TSS) of old and young genes of *Arabidopsis thaliana*.

**Fig. S5:** A bar plot showing the distribution of premature translation termination codons (PTC) in old and young genes of Arabidopsis, rice, and maize.

**Fig. S1**

**
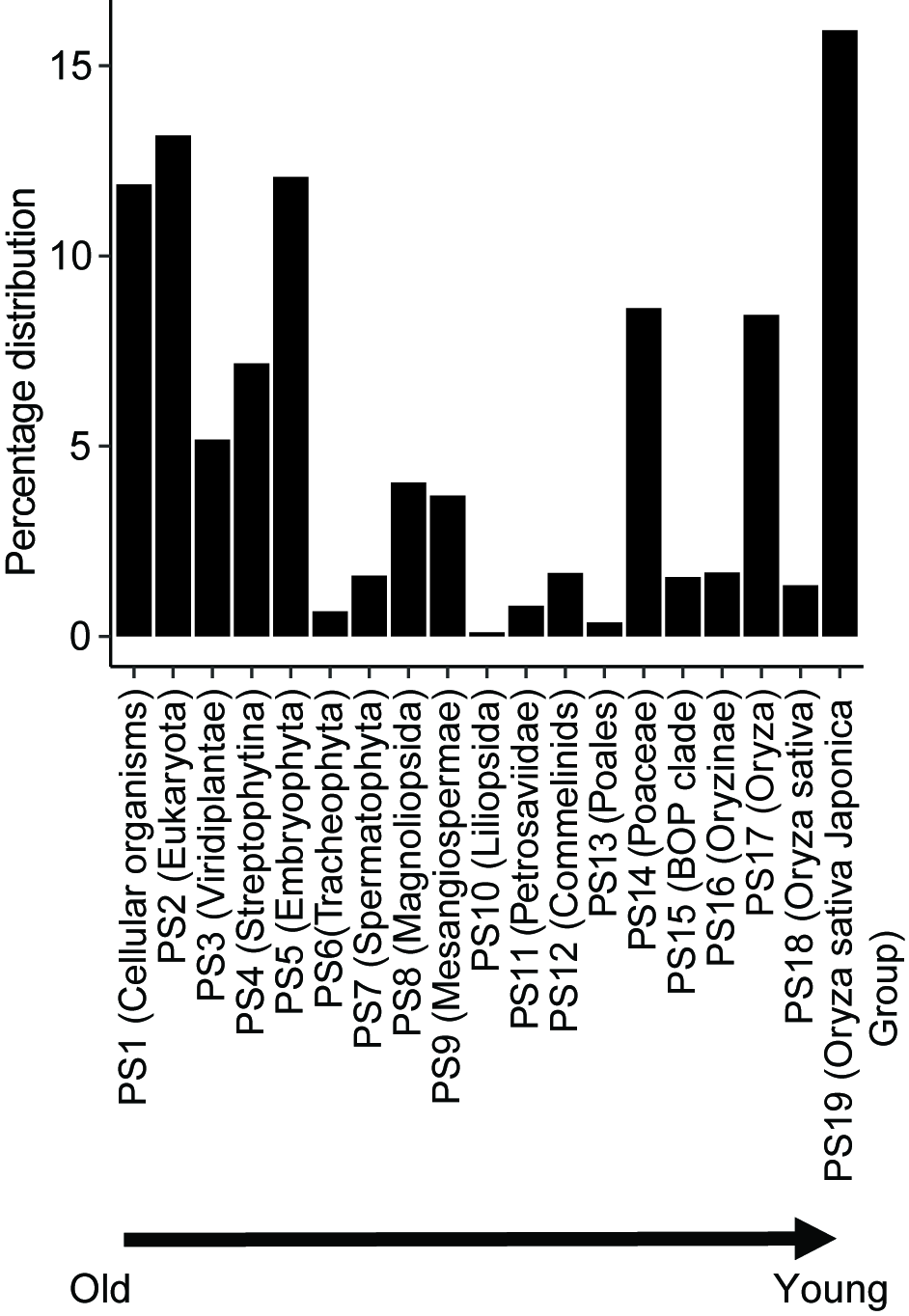
**

**Fig. S2**

**
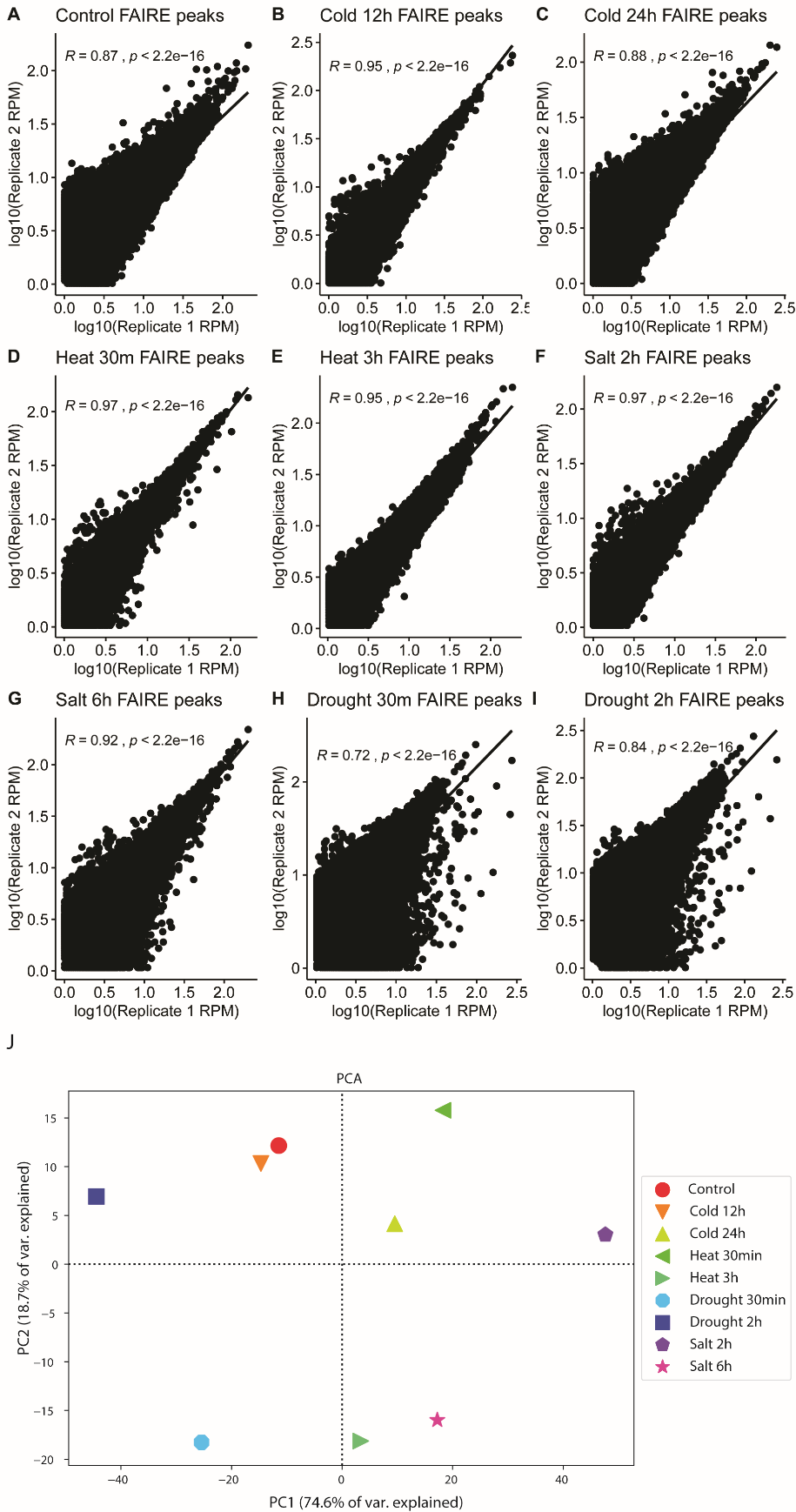
**

**Fig. S3**

**
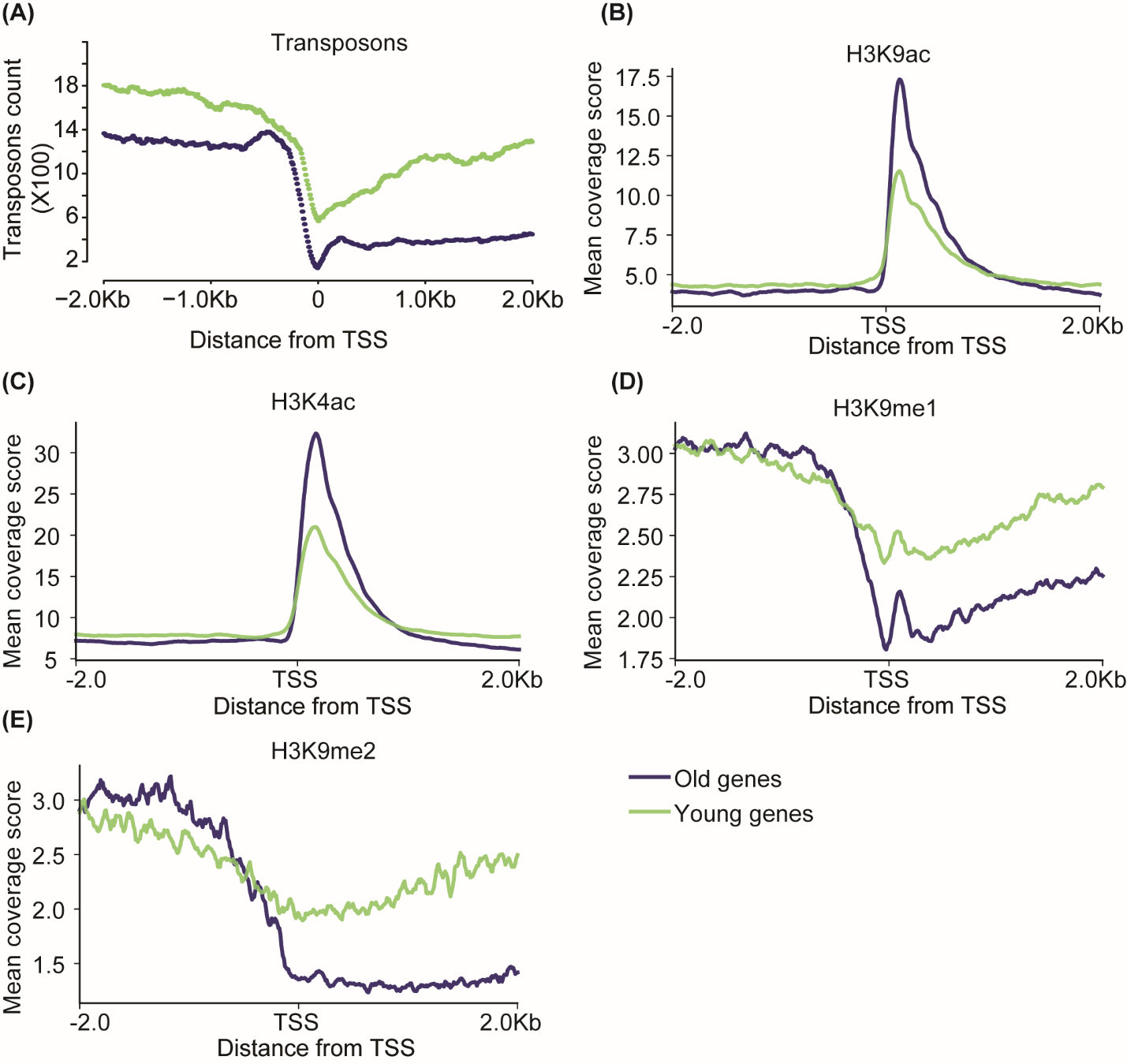
**

**Fig. S4**

**
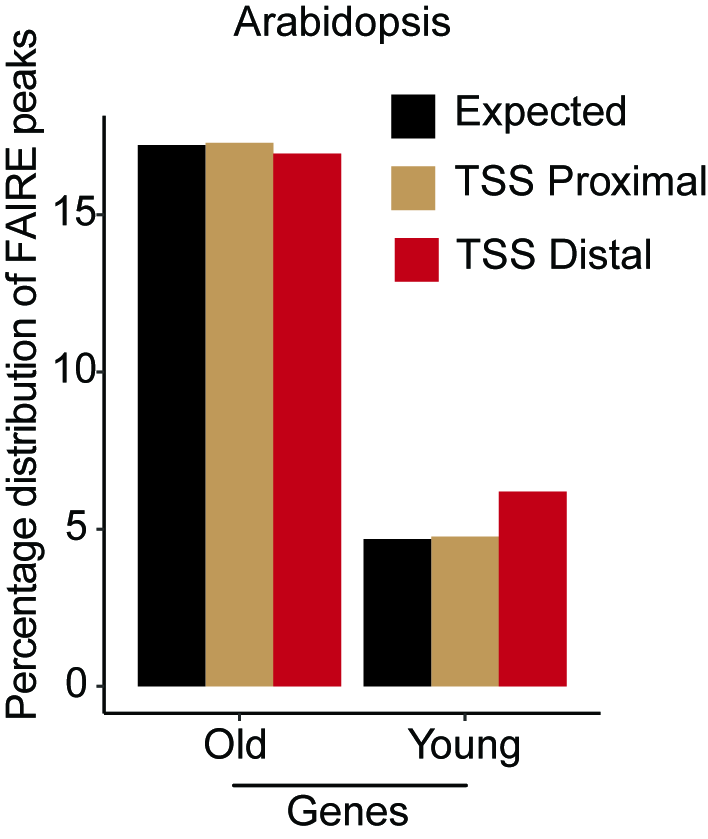
**

**Fig. S5**

**
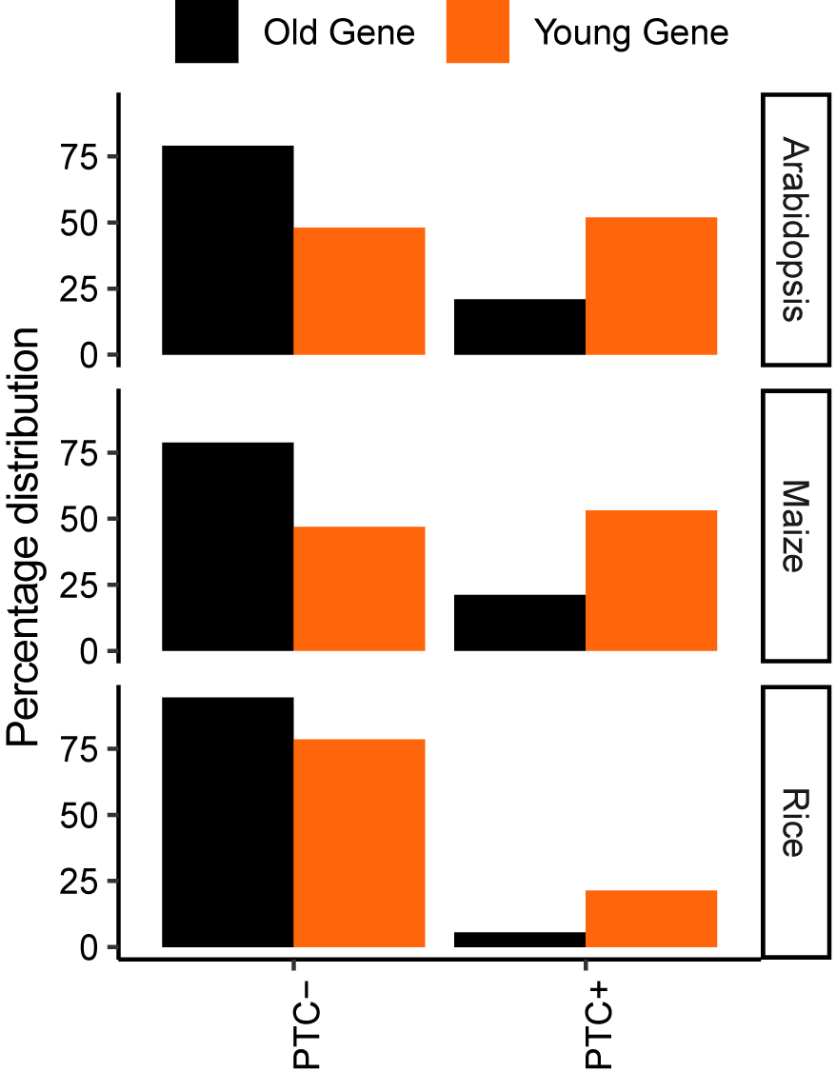
**
